# Analyzing the distribution patterns and dynamic niche of *Magnolia grandiflora* L. in the United States and China in response to climate change

**DOI:** 10.3389/fpls.2024.1440610

**Published:** 2024-10-22

**Authors:** Wenqian Zhang, Xinshuai Wang, Shouyun Shen, Yanghui Zhao, Siwen Hao, Jinghuan Jiang, Donglin Zhang

**Affiliations:** ^1^ College of Landscape Architecture, Central South University of Forestry and Technology, Changsha, Hunan, China; ^2^ Hunan Big Data Engineering Technology Research Center of Natural Protected Landscape Resources, Changsha, Hunan, China; ^3^ Yuelushan Laboratory Carbon Sinks Forests Variety Innovation Center, Changsha, Hunan, China; ^4^ College of Economics and Management, Changsha University, Changsha, Hunan, China; ^5^ Chinese Research Academy of Environmental Sciences, Beijing, China; ^6^ Department of Horticulture, University of Georgia, Athens, GA, United States

**Keywords:** biomod2, introduced species, niche shifts, potential geographic distribution, southern magnolia

## Abstract

**Introduction:**

Magnolia grandiflora L. (southern magnolia) is native to the southeastern coastal areas of the United States, from North Carolina to eastern Texas (USDA Cold Hardiness Zone 8). It is currently widely cultivated in Zones 5-10 in the U.S. and in southern Yangtze River regions in China. Limited studies have examined the effects of climate change and human activities on the geographical distribution and adaptability of M. grandiflora during its introduction to China.

**Methods:**

We selected 127 occurrence points in the U.S. and 87 occurrence points in China, along with 43 environmental variables, to predict suitable habitat areas for M. grandiflora using present climate data (1970-2000) and projected future climate data (2050-2070) based on a complete niche ensemble model (EM) using the Biomod2 package. We also predicted the niche change of M. grandiflora in both countries using the 'ecospat' package in R.

**Results:**

The ensemble models demonstrated high reliability, with an AUC of 0.993 and TSS of 0.932. Solar radiation in July, human impact index, and precipitation of the wettest month were identified as the most critical variables influencing M. grandiflora distribution. The species shows a similar trend of distribution expansion under climate change scenarios in both countries, with predicted expansions towards the northwest and northeast, and contractions in southern regions.

**Discussion:**

Our study emphasizes a practical framework for predicting suitable habitats and migration of Magnoliaceae species under climate change scenarios. These findings provide valuable insights. for species conservation, introduction, management strategies, and sustainable utilization of M. grandiflora.

## 1 Introduction

Over the past 100 years, global climate change and human activities have provided a fertile condition for non-native species to be introduced. The impact of climate change on biodiversity and the development of strategies for conservation have become a serious global issue for the international community. The global climate has experienced significant changes characterized by warming (Craparo et al., 2015). However, there is still a lack of comprehensive understanding of how many introduced plants responding to climate change. Ornamental plants have been domesticated and introduced for thousands of years (Corlett and Westcott, 2013; Pearson and Dawson, 2005; Walck et al., 2011). Species introduction depends on the availability of suitable geographic areas and environmental conditions (Lawler et al., 2009). Long-term field introduction trials have been the most reliable approach to identify suitable areas for woody ornamental plants (Zhang H. N. et al., 2019; Zhang K. et al., 2019). It requires significant resources and observations of several growth periods of plants. Therefore, the prediction of potential distribution pattern changes and ecological niche shifts of plants can be used to explore the potential geographic distribution and ecological niche evolution of plants effectively, which is critical for the study of ecological adaptations and introductions of plants to cultivation. Global climate change and human activities usually provide favorable conditions for the migration and cultivation of species and the distribution range of ornamental plants may increase (Feng et al., 2024; Wu H.-Y. et al., 2024; Wu K. et al., 2024; Zhang et al., 2024). Recent studies aimed to systematically elucidate how environmental variables affect the potential distribution patterns of plants and the transformation of their bioclimatic ecological niches under the pressure of global climate change and human activity (Broennimann et al., 2006; Trew and Maclean, 2021).

Species distribution models (SDMs) serve as an effective way for investigating the relationship between species and environmental variables and for predicting their potential suitable habitats. These models offered a scientific foundation for species conservation and biodiversity protection. Combining environmental variables with species distribution data, SDMs are extensively applied in research on species range shifts under climate change scenarios, conservation efforts, and the prediction of species cultivation ranges in new areas (Booth, 2018; Franklin, 2013; Guisan et al., 2013). The theoretical framework of SDMs is grounded in ecology, geography, and statistics, aiming to predict the spatial distribution of species using known occurrence records and environmental variables (Decocq et al., 2023; Schulze, 2000). However, numerous distribution prediction models have been developed and applied, each with different algorithms and focuses. Consequently, employing various model techniques for the same species can yield divergent results, and a single model may underestimate *M. grandiflora*s’ potential distribution range. Ensemble models (EMs), which combine predictions from qualified single models, have significantly enhanced the accuracy and performance of modeling predictions. One significant issue is that different species require different methods to achieve optimal results, and using the same method for disparate questions is not ideal, particularly in the context of species distribution models (Qiao et al., 2015; Ramasamy et al., 2022). The ability of EMs to accurately predict the cultivation distribution of woody plants remains uncertain, necessitating further research to validate model results and assess the impact of varying environmental variables (Anifowose et al., 2015; Farooq et al., 2021; Shahhosseini et al., 2020). A recent study in Shandong Province, China, attributed the decline of the seagrass system to human activity and climate change. Yang et al. (2023) utilized EMs to predict suitable areas for eelgrass (*Zostera marina*) and implement conservation efforts, demonstrating superior performance of the EM approach compared to single model. Similarly, Kaky and Gilbert (2017) applied EMs to predict the distribution of 114 Egyptian medicinal plants, finding that MaxEnt and Random Forest (RF) outperformed support vector machine (SVM) and classification and regression tree (CART) models. Additionally, Hamid et al. (2019) employed the Biomod2 software package to simulate the distribution of Himalayan birch under climate change, revealing strong robustness in the integrated model.

Climate change is a primary factor influencing species distribution that interacts with habitats of *M. grandiflora*, which is intricately associated with variables such as soil characteristics, land use, human activities, and topography (Li et al., 2013), all of which were pivotal for fulfilling the ecological niche requirements of species (Cramer et al., 2022; Saha et al., 2023). Some researchers, like Velazco et al. (2017), elucidated that the incorporation of both soil and climatic variables as dominated variables in distribution models significantly enhances model accuracy, in stark contrast to models that solely rely on climate prediction variables. At present, there exists a lack of consensus regarding the relationship between dynamic niche shifts of native and introduced species and environmental variables, thereby underscoring the imperative need for further exploration of this dynamic niche status (Atwater et al., 2018; Early and Sax, 2014; Richardson and Pyšek, 2012). The climatic niche of a species consisted of the fundamental niche, depicting the optimal environmental conditions a species can inhabit, unaffected by factors like competition and predation (Peterson et al., 2017), alongside the realized niche, which is a restricted subset of the fundamental niche due to limiting variables (Araújo et al., 2013; Pulliam, 2000). Certain scholars advocated for niche shift based on an ecological niche expansion index exceeding 10% (Soberón and Arroyo-Peña, 2017), suggesting that during the transition of niche shift from native to introduced areas (Anderson, 2013), *M. grandiflora*s predominantly occupied the introduced area that were not included in the native area (Bates et al., 2020; Petitpierre et al., 2012; Torres et al., 2018). Conversely, other researchers have emphasized the use of the unfilled niche index to analyze whether an introduced area possesses analogous climate conditions not currently occupied by *M. grandiflora* in its native range due to environmental constraints and limiting variables. This approach provides critical insights into the optimal conditions for species survival (Ashby et al., 2017; Polidori et al., 2018; Walker and Valentine, 1984). This analysis holds significant importance in comprehending the ecological adaptability of ornamental woody plants and in informing strategies related to their introduction and cultivation.

Southern magnolia is a member of the *Magnolia* family, which originates from the coastal areas of the southeastern US. It can reach a height of 8–30 m and is widely used as an ornamental woody plant, with an elegant and majestic architecture, dense leaves, and large white fragrant flowers. *M. grandiflora* is an aristocratic broadleaf evergreen that began to be cultivated in the United States in 1734 due to its significant landscape value (Dirr, 1998). The native range of *M. grandiflora* extends from eastern North Carolina, south along the Atlantic Coast to the Peace River in central Florida, then westward through roughly the southern half of Georgia, Alabama, and Mississippi, and across Louisiana into southeast (Little, 1979; Sokkar et al., 2014). It is widely cultivated around the world, especially in the southern area of the Yangtze River in China (Greller, 1989; Li et al., 2015), which is the most successful ornamental woody plant in *Magnoliaceae* that have been introduced from the US to China (Vastag et al., 2020). The introduction of *M. grandiflora* is essentially a dynamic process of species dispersal and distribution (Gruhn and White, 2011; Hoyle et al., 2017). Its potential geographic distribution is highly dependent on its adaptation to the new environmental conditions (Berger et al., 2007). Consequently, it is imperative to investigate how *M. grandiflora* adapts to climatic shifts and to assess its adaptability and distribution during introduction and cultivation in China. Some researchers have explored the ecological niche alterations and potential distribution of cultivated *M. grandiflora* (Farag and Al-Mahdy, 2012; Wang, 2006), analyzing its potential expansion range by comparing the climatic environments of introduced areas (China) with its native habitat (USA). Such analyses are critical for devising effective prevention and control strategies by predicting spatiotemporal diffusion characteristics and ecological niche shifts (Mitchell and Power, 2003). Current research on *M. grandiflora* primarily concentrated on ecological characteristics (Li et al., 2013), physiological responses to drought stress (Sukumaran et al., 2020), medicinal value (Lee, 2011; Xu et al., 2024), landscape design, and economic value (Ali et al., 2022). However, there is a lack of analysis on suitable cultivation areas for *M. grandiflora* and research on its niche shifts. At present, there is a lack of research addressing potential distribution pattern changes and niche shifts of *M. grandiflora* between its native and introduced regions (Knox et al., 2012). There are only references to the expansion of *M. grandiflora* in North Carolina Piedmont forest; more studies focused on biogeographic patterns between bacterial phyllosphere communities of *M. grandiflora* at present (Gruhn and White, 2011; Stone and Jackson, 2016; Wang et al., 2022). To fill this gap, we established an ensemble model utilizing environmental variables such as bioclimate, topography, soil, NDVI, and human activities. Our aim is to predict suitable distribution areas of *M. grandiflora* and evaluate the dynamic ecological niche between its native of the USA and introduced areas of China, exploring the existence of niche shifts and their primary influencing variables. Based on our research findings, we propose a scientifically informed strategy for the conservation and introduction of *M. grandiflora.*

## 2 Materials and methods

### 2.1 Occurrence data of *M. grandiflora*

To obtain comprehensive geographical distribution information of *M. grandiflora* in the USA and China, we employed three methods. First is field investigation. From 2019 to 2023, we carried out field surveys of natural populations in the USA, documenting the latitude and longitude of each plot and habitat characteristics. In China, we also collected distribution data from some cultivated *M. grandiflora* with health and vigorous growth. Second are species occurrence databases. We extracted distribution points of *M. grandiflora* from various species distribution websites, including Global Biodiversity Information Facility (GBIF), iplant (www.iplant.cn), SouthEast Regional Network of Expertise and Collections (SERNEC, https://sernecportal.org/portal/index.php), the Forest Research Institute NSII (China National Specimen Information Infrastructure), and China Virtual Herbarium (CVH). Third are scientific articles. We conducted searches for *M. grandiflora* with Latin name in the Web of Science (WOS; https://www.webofscience.com/) and China National Knowledge Infrastructure (CNKI; https://www.cnki.net/) databases, retaining literature with precise records of field distribution locations.

**Figure 1:**
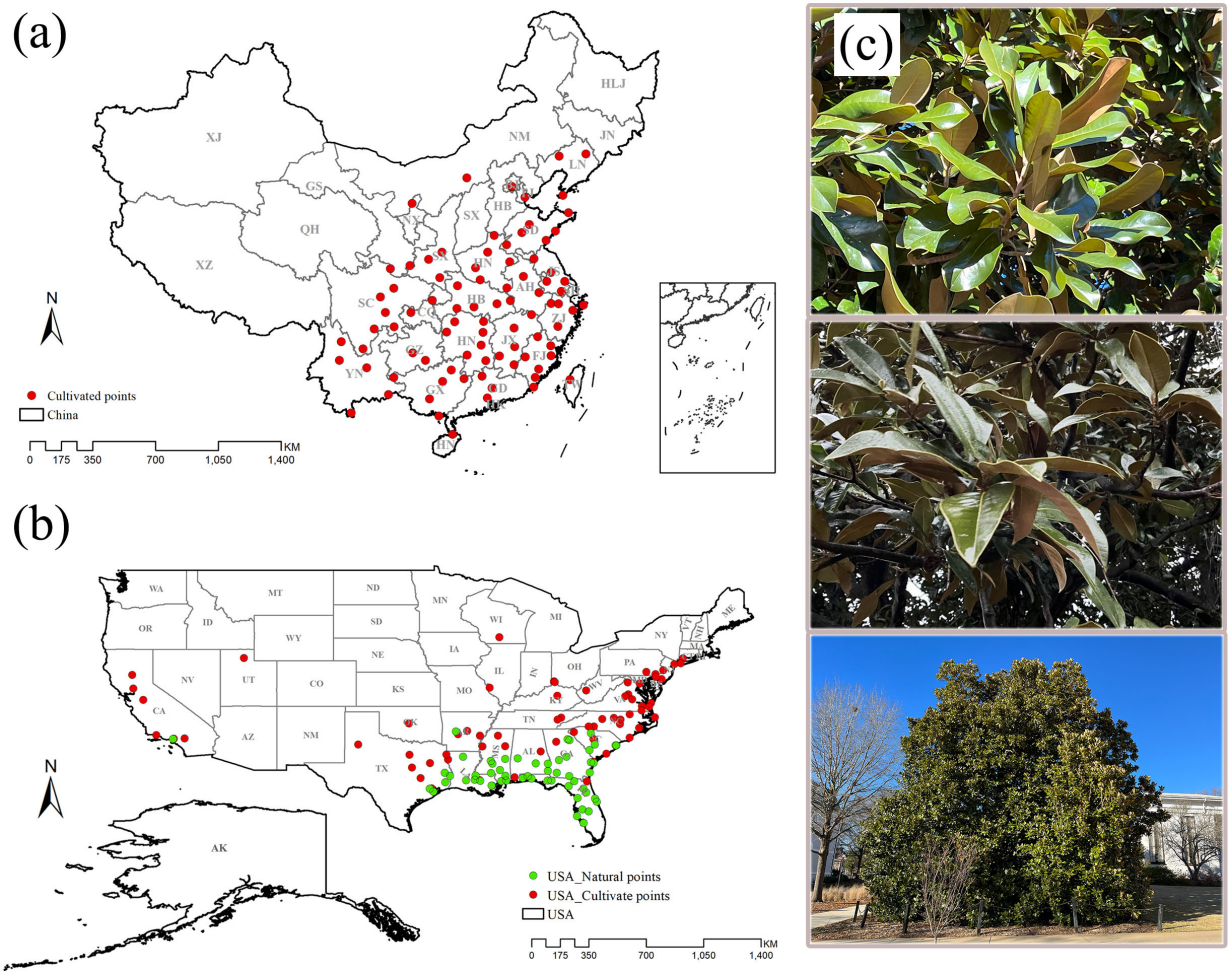
Spatial distribution of occurrence points of *M. grandiflora* in China and the USA. **(A)** Occurrence points (87 points) in China; **(B)** occurrence points (127 points) in the USA. Red is the cultivated distribution points, and green is the natural distribution points. **(C)** The M. grandiflora observed during the field survey is found in the USA.

In ensuring the accuracy and timeliness of *M. grandiflora* distribution points that we collected, we focused on two dimensions: spatial and temporal. Spatially, the collected species distribution points had to correspond to specific villages or streets. Regarding the temporal scale, we restricted our analysis to species distribution points that were collected in the year 2000 or later. We converted specific locations into latitude and longitude using Google Earth. Adhering to these criteria, we obtained a total of 236 occurrence points for *M. grandiflora*, comprising 134 occurrence points in the USA and 102 occurrence points in China. However, to address spatial autocorrelation among the collected distribution points, we initially utilized ENM tools to ensure that only one valid species distribution point existed within each environmental variable grid of 2.5 arcmin (~5 km) (Warren et al., 2010). Subsequently, we established a buffer zone with a radius of 150 km around each point using ArcGIS 10.3.0 (Greaves et al., 2006). These measures were implemented to enhance the robustness of the statistical analysis. The final dataset for subsequent analysis included 127 distribution points of *M. grandiflora* in the USA (56 natural occurrence points and 71 cultivated occurrence points) and 87 occurrence points in China (**Figure 1**). We downloaded administrative boundary maps of China and the USA from http://www.gadm.org/country.

### 2.2 Selection and comparison of environmental variables

Based on previous research, habitat characteristics of *M. grandiflora* and expert recommendations for the *Magnoliaceae* (Delcourt and Delcourt, 1977; Quarterman and Keever, 1962; Wang et al., 2022; White, 1987), we selected 43 environmental variables, including bioclimatic variables, topography, soil, NDVI, anthropogenic activities, and other environmental variables, to construct an ecological dataset (**Supplementary Table S1**).

Consistent with previous studies of vegetation dynamics (Li et al., 2019; Yang et al., 2021), we employed predictions from general circulation models (GCM) under three future greenhouse gas emission scenarios, SSP1-2.6, SSP.3-7.0, and SSP5-8.5 scenarios to assess the influence of climate change on species distribution. We opted for the BCC-CSM2-MR model, which is extensively utilized in the Middle East Asia region and exhibits superior simulation capabilities for the East Asian climate, particularly temperature, in comparison to other climate models (Chen et al., 2022; Wu, 2020). Climate factor raster data were acquired from the WorldClim database (www.worldclim.org) which interpolates the monthly average values of 30 standard years of bioclimatic variables from 1970 to 2000 to generate global climate raster data (2.5 arcmin). We utilized ArcGIS 10.3.0 to 19 bioclimatic variable layers by the extent of China (Wertz & Wilczyński, 2022). The solar radiation index (SRAD) was also obtained from the WorldClim database (Gunawan et al., 2021). The potential evapotranspiration (PET) was derived from the Global-AI_PET_v3 database, based on the third edition of the Global Aridity Index and Potential Evapotranspiration (Zomer et al., 2022). The Penman–Monteith reference evapotranspiration (ET0) equation, based on the Food and Agriculture Organization (FAO), was used to provide global high-resolution hydroclimatic monthly and annual average data (1970–2000). The digital elevation model (DEM) data were provided by the International Science Data Service Platform of the Chinese Academy of Sciences (ISDSP, http://datamirror.csdb.cn/ (Han et al., 2018), from which slope and aspect data were generated by ArcGIS 10.3.0. Soil variables included five variables related to soil physical and chemical properties and classification: organic carbon density, sand, silt, nitrogen, and pH water, sourced from SoilGrids (https://soilgrids.org/ (Hengl et al., 2014; Hengl et al., 2017) Considering the actual length of *M. grandiflora* root growth, we selected the arithmetic mean of the top 5 layers (30–60 cm) of soil profiles relevant to *M. grandiflora* growth for modeling. The normalized difference vegetation index (NDVI) was obtained from MODIS/Terra Vegetation Indices 16-Day L3 Global 250 m SIN Grid (2020) (Ramírez-Cuesta et al., 2021). Human footprint (HFP) and human influence index (HII) were obtained from the internet (http://sedac.ciesin.columbia.edu/wildareas/) (Luo et al., 2016), indicating cumulative human pressure on the environment. The resolution and coordinate system of environmental variables were standardized and unified using the Resample function in ArcGIS 10.3.0, with a resolution of 2.5 arcmin. Due to the high autocorrelation with bioclimatic variables, which may lead to overfitting of SDMs, we successfully extracted precise environmental values at species distribution points from the environmental raster data using ArcGIS 10.3.0 (WGS_1984). We used Pearson correlation analysis and VIF to select variables that were less correlated with other variables (∣P∣<0.8, VIF<10) and statistically significant (Pradhan, 2016). Finally, 20 variables were retained.

### 2.3 Model evaluation and selection

We used Biomod2 package in RStudio 4.2.1 to apply 10 model algorithms, GLM, GBM, GAM, CTA, ANN, SRE, FDA, MARS, RF, and MaxEnt, to predict the potential distribution range of *M. grandiflora* in the USA based on its native distribution data and global environmental variables. Pseudo-absence points were generated using a “random” method to create 1,000 background points required for species distribution modeling. For each model, parameters were randomly selected with 75% of the distribution records used as the training datasets and the remaining 25% as the testing datasets. The data were divided into training and testing sets five times, with modeling repeated 10 times. The true skill statistics (TSS) and receiver operating characteristic (ROC) curve were considered key variables for assessing the accuracy of the models. Based on the area under the ROC curve (AUC) value, model performance was categorized as excellent (AUC > 0.9), good (0.8–0.9), fair (0.7–0.8), or poor (0.6–0.7). Additionally, the TSS was used to evaluate the model’s performance, with models classified as excellent (TSS > 0.8), good (0.6-0.8), fair (0.4–0.6), poor (0.2–0.4), or unacceptable (TSS < 0.2). Therefore, we selected excellent models with AUC and TSS values both > 0.9 as candidate models (Nhu et al., 2020). After evaluating the accuracy of the 10 models, we identified three models, MARS, MaxEnt, and RF, with average TSS and ROC values exceeding 0.95. Subsequently, we decided to use these three models to construct an ensemble model (EM) for predicting the potential suitable of *M. grandiflora* in native and introduced areas in 2050 and 2070.

### 2.4 Suitable area classification and distribution pattern changes

For better visualization of the potential suitable habitats and distribution pattern changes of *M. grandiflora*, we processed the output ASCII grids from the “biomod2” package using the natural break method in the Reclassify function of ArcGIS 10.3.0. The suitability levels were categorized into four groups: unsuitable area (0.0–0.2), low suitability area (0.2–0.4), moderate suitability area (0.4–0.6), and high suitability area [0.6–1.0 (Jung et al., 2016)]. The criteria for identifying the areas of distribution pattern change involved using the Raster Calculator in ArcGIS 10.3.0 to subtract and overlay the climate change scenarios of each period with the current scenario, resulting in four categories: 0→0 unsuitable area; 0→1 expansion area; 1→0 loss area; and 1→1 stable area (Miettinen et al., 2016).

### 2.5 Data analysis and niche dynamics

**Figure 2:**
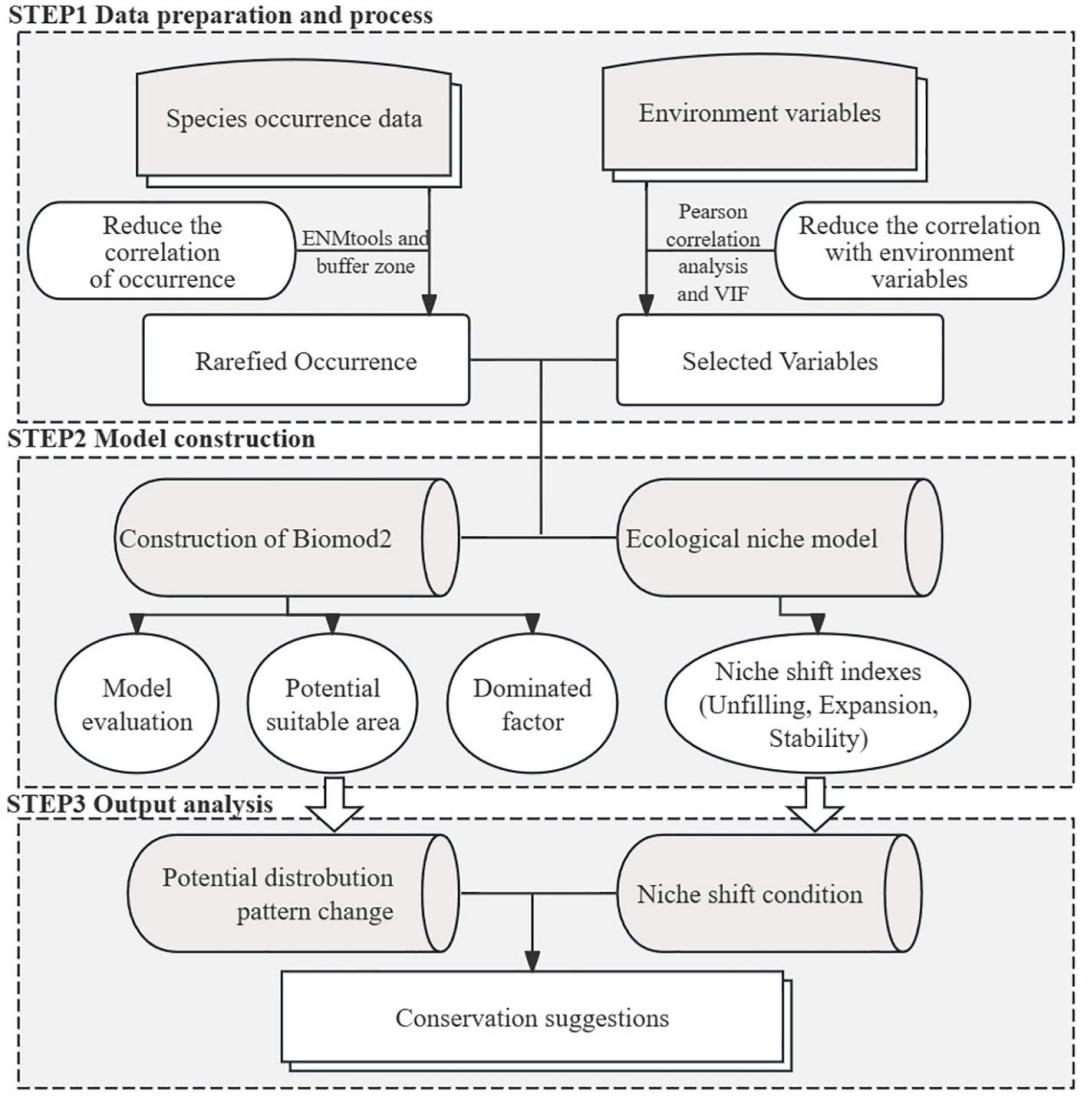
The technical frame of this study. It is divided mainly into data preparation and process, model construction, and output analysis.

The conservatism of the climatic niche between the native range (USA) and the introduced range (China) was validated using the “ecospat” package and the COUE scheme (Di Cola et al., 2017). First, a minimum convex polygon containing 5,000–10,000 background points was created, and the “Wallace” package was used to surround the distribution points of *M. grandiflora* in each country (Kass et al., 2023). Then, the background point values of each climatic variable grid were extracted, and a principal component analysis (PCA-env) of the native and introduced ranges was conducted. The kernel density function was used to standardize and concentrate occurrence points and background points, effectively reducing sampling bias (Monsarrat et al., 2019; Palpanas et al., 2003). Based on the training threshold and specificity threshold of the ensemble model, suitable areas (1) and unsuitable areas (0) were determined in the basic statistical analysis. The purpose of similarity testing and equivalency testing was to examine, through 100 repetitions of training and calculation, whether there were similar climates that could be occupied by *M. grandiflora* in the future and whether the specific form of climate conditions in the introduced range was equivalent to that of the native range. The overlap of the ecological niche of *M. grandiflora* in the native and introduced ranges was calculated using Schoener’s D (Schoener, 1968) and Hellinger’s distance (I) (Warren et al., 2011). The range of values for D and I is [0 (no overlap), 1 (complete overlap)] (**Figure 2**).

## 3 Results

### 3.1 Model accuracy evaluation for simulating the potential suitable area

**Figure 3:**
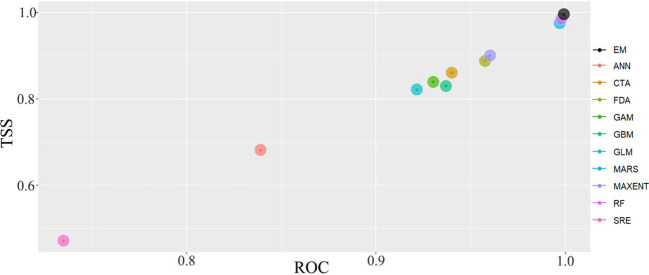
Comparison of AUC and TSS between single and ensemble model. EM, ensemble model; MaxEnt, maximum entropy model; RF, random forest; MARS, multivariate adaptive regression splines.

Based on the biomod2 platform in the R, the ensemble model (MAXENT, RF, MARS) with AUC and TSS values exceeding 0.950 was selected. The prediction accuracy of the ensemble model indicates that the ROC of MARS, MaxEnt, and RF are all >0.960, and TSS are all >0.900. The average ROC is 0.993, and the average TSS reaches 0.932, demonstrating extremely high rationality and credibility in predicting the potential distribution of *M. grandiflora* (**Figure 3**).

### 3.2 Testing for ecological niche conservatism

**Figure 4:**
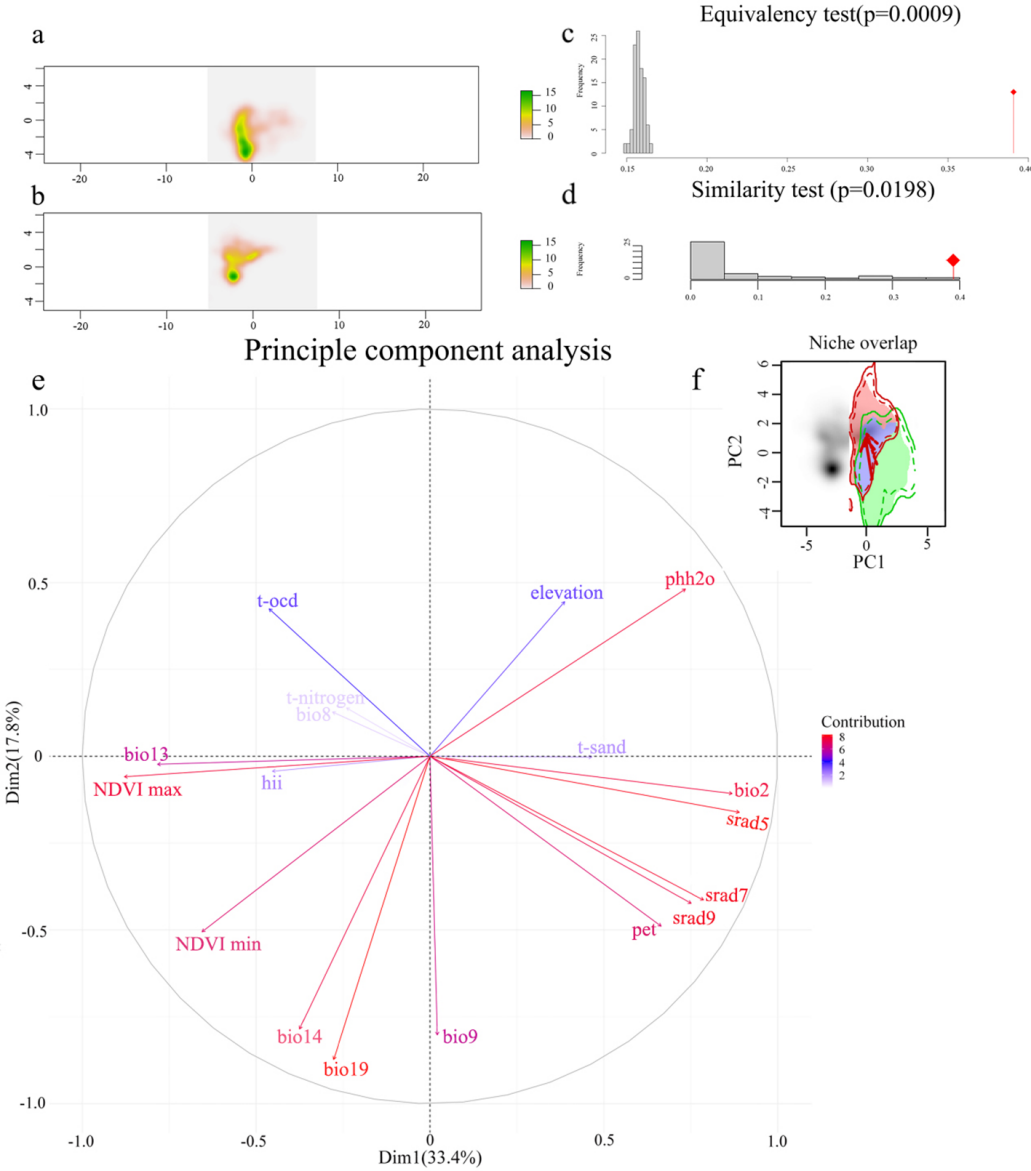
Niche comparison analysis between native and introduced area. Note: The photo above shows the distribution of environmental density in the two-dimensional space of available environments for environmental density distribution in two-dimensional available environmental space within the **(A)** native range and **(B)** introduced range. Red tones indicate environments with lower occurrences, while yellow and green tones indicate environmental conditions that are more commonly occupied. **(C)** Similarity test (p = 0.0009) and **(D)** equivalency test (p = 0.0198). **(E)** Ranking plot of the top two principal components generated in the PCA, where red color indicates a higher contribution of the variable, and blue color indicates a lower contribution of the variable. Upper right corner **(F)** indicates changes in ecological niche dynamics. Ecological niche overlap between the native area USA and the introduced area China. Green shading indicates unfilling areas in the native area, purple indicates niche overlap, and red indicates niche expansion in the introduced areas. The solid and dashed contour lines illustrate 100% and 50% of the available environmental space. The direction of the solid red arrow is a shift in the center of species density, and the direction of the dashed red arrow is a shift in environmental space.

This study visualized the environmental conditions between the native habitat in the USA and the introduced habitat in China through principal component analysis (PCA) of 20 environmental variables. PC1 explained 17.8% of the selected variables, while PC2 explained 33.4%, with a total explanation rate of 51.2% (**Figure 4E**). The first principal component axis, PC1, was positively correlated with May solar radiation and negatively correlated with maximum annual normalized difference vegetation index. The second principal component axis, PC2, was negatively correlated with precipitation in the coldest quarter and positively correlated with water body pH. We also observed a shift in the ecological niche of *M. grandiflora* towards decreasing PC1 and increasing PC2 (**Figure 4F**), indicating a preference for locations with lower May solar radiation and higher maximum annual normalized difference vegetation index PC1 and higher water body pH and lower precipitation in the coldest quarter PC2. The density of species occurrence within environmental space is illustrated in **Figures 4A, B**. The results suggested that the migration trend of *M. grandiflora* in its native habitat was similar to that of the introduced habitat, with a high stability value of 0.786 and a low expansion value of 0.213. However, the ecological niche overlap between the native and introduced habitats is low [Schoener’s D = 0.39, Hellinger’s distance (I) = 0.467, where 0 indicates no overlap and 1 indicates complete overlap]. According to the theory of ecological niche conservatism, the null hypothesis is accepted when p-values are all <0.05. The observed ecological niche overlap is outside the 95% confidence interval, indicating that the ecological niche forms, sizes, and spatial characteristics of *M. grandiflora* in its native and introduced habitats are not identical. Excluding experimental randomness, the observed ecological niche overlap in the ecological niche similarity test (**Figure 4C**) fell within the 95% confidence interval, suggesting that there were more similar climate regions between the native and introduced habitats. The results of the equivalence and similarity tests (**Figure 4D**) indicated that although the dynamic ecological niches of the native habitat were not identical to those of the introduced habitat, there was a large unfilled value in the ecological niche (0.620), indicating a shift of the ecological niche towards climatically similar conditions between the native and introduced habitats.

### 3.3 Environmental variables’ responses to and contribution rates on the ecological niche of *M. grandiflora*

**Figure 5:**
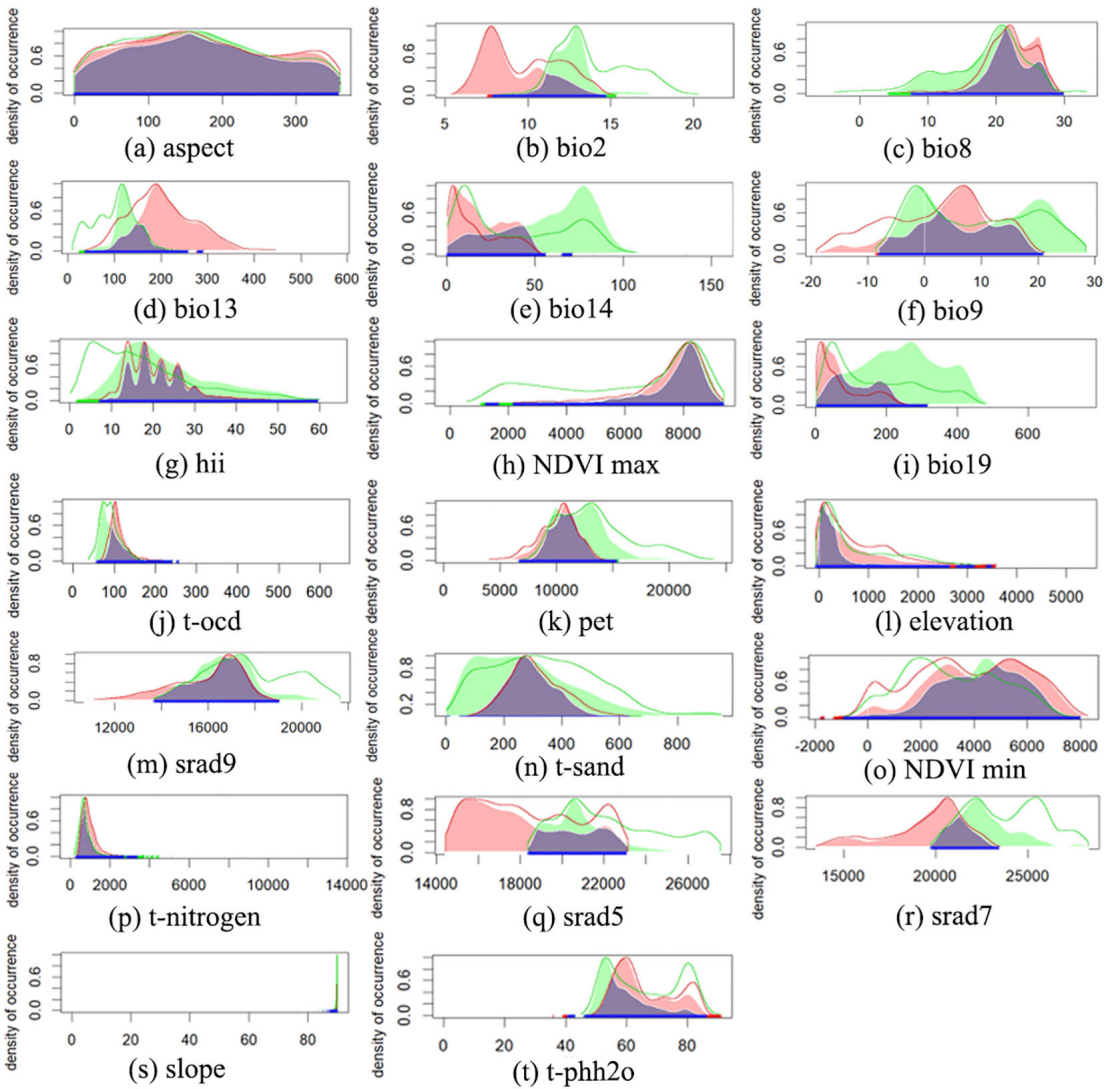
Predicted niche occupancy (PNO) curves. Blue solid lines indicate predicted niche Overlap, green solid lines indicate predicted native area niche, and red solid lines indicate predicted introduced area niche. Peaks of overlap indicate similar climatic tolerances, and the width of the profile indicates the specificity of climatic tolerances. Green and red solid contours represent 100% of the available environmental space for native and introduced area, respectively. **(A)** Aspect, **(B)** mean diurnal range (Bio2), **(C)** mean temperature of wettest quarter (Bio8), **(D)** precipitation of wettest month (Bio13), **(E)** precipitation of driest month (Bio14), **(F)** mean temperature of driest quarter (Bio9), **(G)** human influence index (HII), **(H)** maximum normalized difference vegetation index (NDVI max), **(I)** precipitation of coldest quarter (Bio19), **(J)** organic carbon density (t-ocd), **(K)** potential evapo-transpiration (pet), **(L)** elevation, **(M)** solar radiation in September (srad 9), **(N)** sand (t-sand), **(O)** minimum normalized difference vegetation index (NDVI min), **(P)** nitrogen (t-nitrogen), **(Q)** solar radiation in May (srad 5), **(R)** solar radiation in July (srad 7), **(S)** slope; **(T)** pH water (t-phh2o).

**Figure 6:**
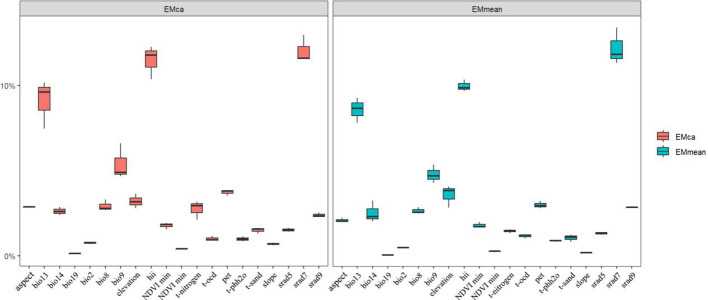
Importance ratios of environmental factors in the ensemble model. Emca and emmean are two different algorithms in the ensemble model, the horizontal axis is the short name of the environmental factors in turn, the vertical axis is the size of the percentage of the importance of the environmental factors during the model run, and the box-and-line plot represents the average of 10 modeling runs.

The predictive niche occupancy (PNO) curve (**Figure 5**) demonstrated that between its native and introduced ranges, the niche of *M. grandiflora* was sensitive to changes in variables such as temperature, precipitation, water pH, and solar radiation. In contrast, variables such as topography, human activities, NDVI, and soil texture have a smaller impact on its niche shift, as indicated in the principal component analysis (**Figure 4E**), with the variables contributing less plotted in blue. The relative importance of environmental variables in the ensemble model was indicated (from **Figure 6**); July solar radiation (2,400–2,500), Human Footprint Index (19.5–26.1), and maximum wet month precipitation (128.2–240.0 mm) were the top 3 variables affecting its distribution. The influence of the driest season’s mean temperature (−10°C–29.9°C), potential evapotranspiration (8,000–11,000), and elevation (0–250 m) was relatively weaker.

### 3.4 The distribution of *M. grandiflora* under the current climate

**Figure 7:**
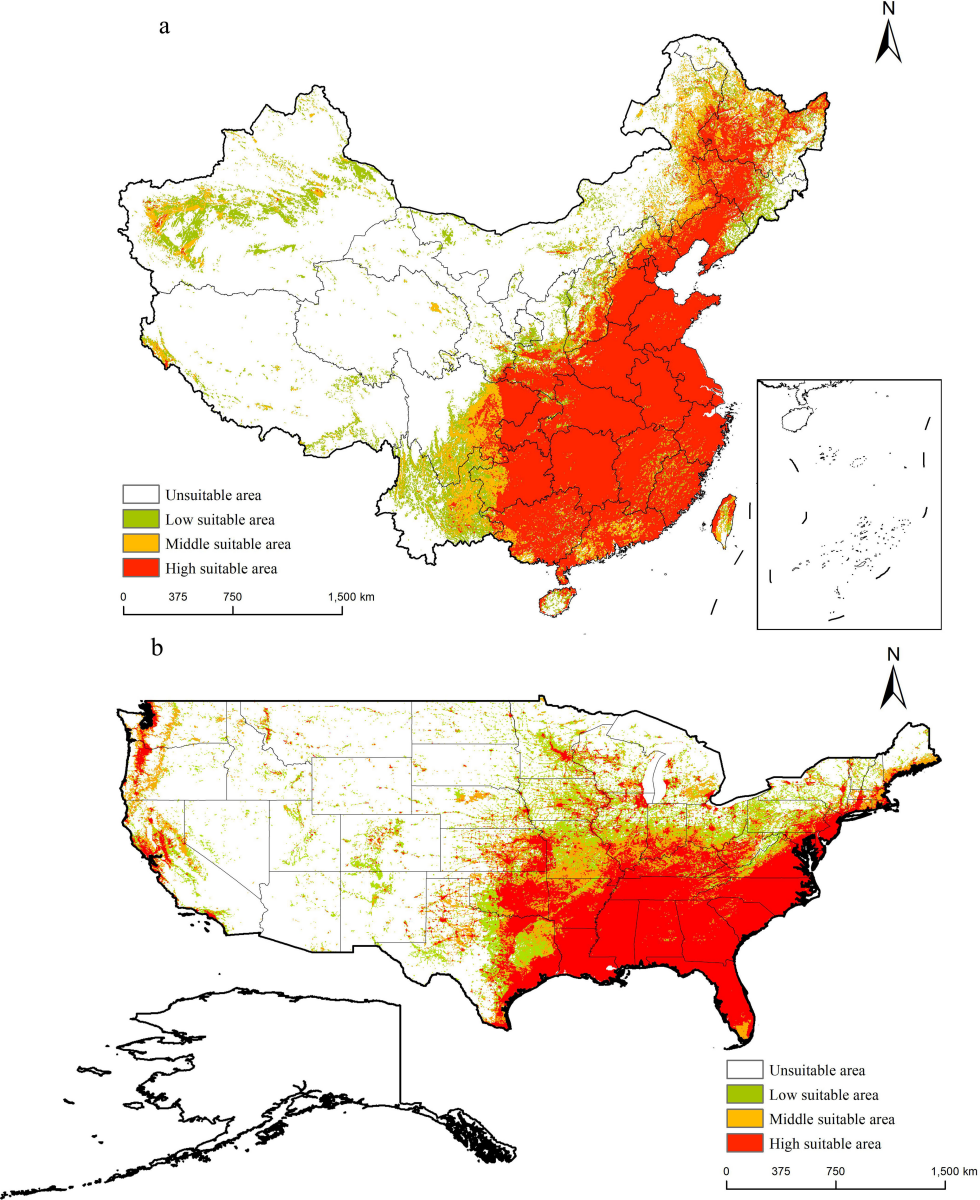
Suitable areas of *M. grandiflora* in China and the USA under the current climate conditions. **(A)** China; **(B)** the USA. Note: White on the map indicates non-suitable areas, green indicates low suitable areas, orange indicates medium suitable areas, and red indicates high suitable areas.

The potential distribution area of *M. grandiflora* (**Figure 7**) indicated that it is primarily distributed in eastern China. High suitability areas include Beijing, Tianjin, Hebei, southern Shanxi, Henan, Shandong, southern Shaanxi, Anhui, Jiangsu, Shanghai, eastern Sichuan, Chongqing, eastern Guizhou, Guangxi, Guangdong, Fujian, Zhejiang, Hubei, Hunan, Jiangxi, northern Hainan, and Taiwan. Moderate suitability areas surround these high suitability regions and extend to northeastern Inner Mongolia, northern Beijing, northern Hebei, southeastern Shanxi, eastern Sichuan, western Guizhou, eastern Yunnan, southern Guangxi, and central Guangdong. Additionally, the western moderate suitability areas include southern and southwestern Xinjiang and northeastern Qinghai. Low suitability areas are found in eastern Jilin, eastern Liaoning, northern Shanxi, northern Yunnan, northern Inner Mongolia, eastern Tibet, and Xinjiang.

In the USA, a similar distribution pattern is observed. High suitability areas are concentrated in Florida, eastern Texas, Louisiana, Mississippi, Alabama, Georgia, South Carolina, Tennessee, Arkansas, Virginia, Kentucky, southern Illinois, southern Indiana, Missouri, Oklahoma, eastern Kansas, Connecticut, Delaware, Maryland, Massachusetts, New Jersey, New York, Pennsylvania, Minnesota, California, Oregon, and Washington. Moderate suitability areas include southern Florida, southern Maine, Connecticut, Massachusetts, Rhode Island, northern Arkansas, southern Illinois, southern Indiana, southern Michigan, Missouri, northern Nebraska, southern Ohio, and northeastern Texas. Low suitability areas are found in Colorado, Illinois, Indiana, Iowa, Minnesota, Missouri, New Mexico, Ohio, Oklahoma, Pennsylvania, Texas, Utah, Virginia, and West Virginia.

### 3.5 Future changes in suitable habitat areas of *M. grandiflora*

This study compared the potential distribution areas of *M. grandiflora* under climate change in China and the USA. For China, projections for 2050 indicate that low suitability areas will initially increase, followed by a decrease, while moderate and high suitability areas will show a continuous upward trend. By 2070, low suitability areas are expected to first decrease and then increase, while moderate suitability areas will continue expanding. Notably, high suitability areas are projected to reach their peak in the 2070-SSP585 scenario, covering 210.46×10^4^ km^2^. In contrast, while the total suitable area in China shows continuous growth by the 2070s, the trend in 2050 is characterized by fluctuating increases.

For the USA, in 2050, low and moderate suitability areas will first increase and then decrease, reaching their maximum extent in the 2050-SSP370 scenario at 87.40×10^4^ km^2^ and 78.39×10^4^ km^2^, respectively. Unlike the trends observed in the low and moderate suitability areas, the high suitability area and total suitable area will fluctuate but still show an average increase of 26.75×10^4^ km^2^ and 29.41×10^4^ km^2^ compared to the current suitable areas. By the 2070s, low and moderate suitability areas in the USA are projected to continue increasing, reaching maximum values of 98.06×10^4^ km^2^ and 87.68×10^4^ km^2^, respectively, while the high suitability area and total suitable area are expected to first increase and then decrease.

**Figure 8:**
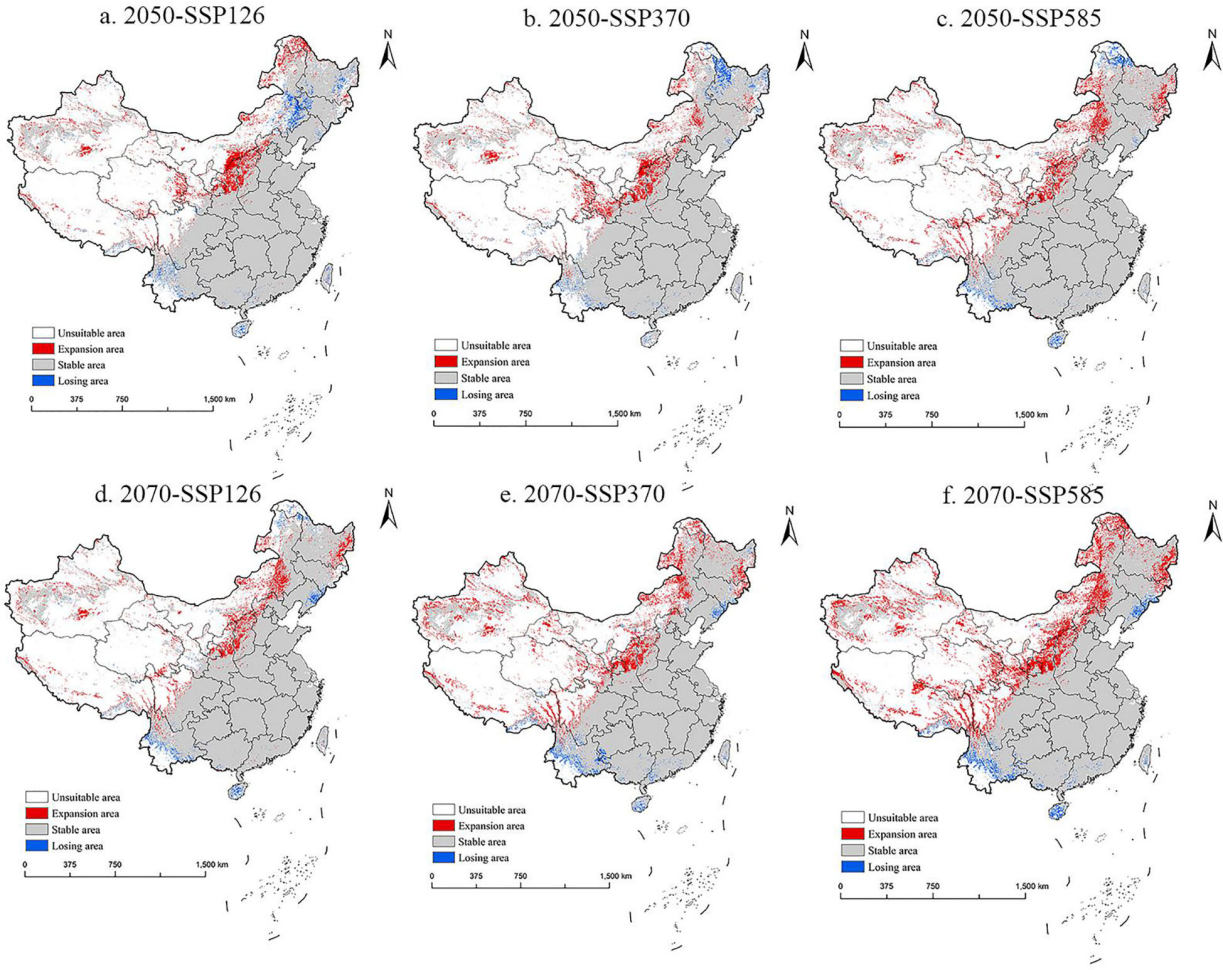
The potential distribution pattern of *M. grandiflora* in China under future climate scenarios (in the 2050s and 2070s). **(A)** 2050-SSP126, **(B)** 2050-SSP370, **(C)** 2050-SSP585, **(D)** 2070-SSP126, **(E)** 2070-SSP370, **(F)** 2070-SSP585. Note: White, red, gray, and blue represent non-suitable area, expansion area, stable area, and losing area, respectively.

The future potential distribution trend of *M. grandiflora* in China is shown in **Figure 8**. In 2050, the suitable area for *M. grandiflora* is projected to expand from the Qinghai–Tibet Plateau to the northwest of China, particularly in regions such as Shanxi, Shaanxi, southeastern Gansu, eastern Qinghai, and even parts of Xinjiang and Xizang. Additionally, there is slight diffusion into northeastern areas, including northeastern Inner Mongolia, eastern Heilongjiang, and the border areas of Jilin. Correspondingly, in 2050, the suitable area will expand from China Cold Hardiness Zones 4a–8b, while the shrinking areas will primarily be in northern China within Cold Hardiness Zones 1a–4a. The diffusion trend in 2070 mirrors that of the 2050s but is more pronounced. Across all climate scenarios, the areas of loss are scattered across southern China, including Hainan, Yunnan, southern Xizang, and Taiwan. There is also a decrease in some northeastern regions, such as northern Heilongjiang and northeastern Inner Mongolia, in 2050. By 2070, *M. grandiflora* is expected to expand across Cold Hardiness Zones 3a–10a, with shrinking areas mainly in southern China within Zones 10b–13b.

**Figure 9:**
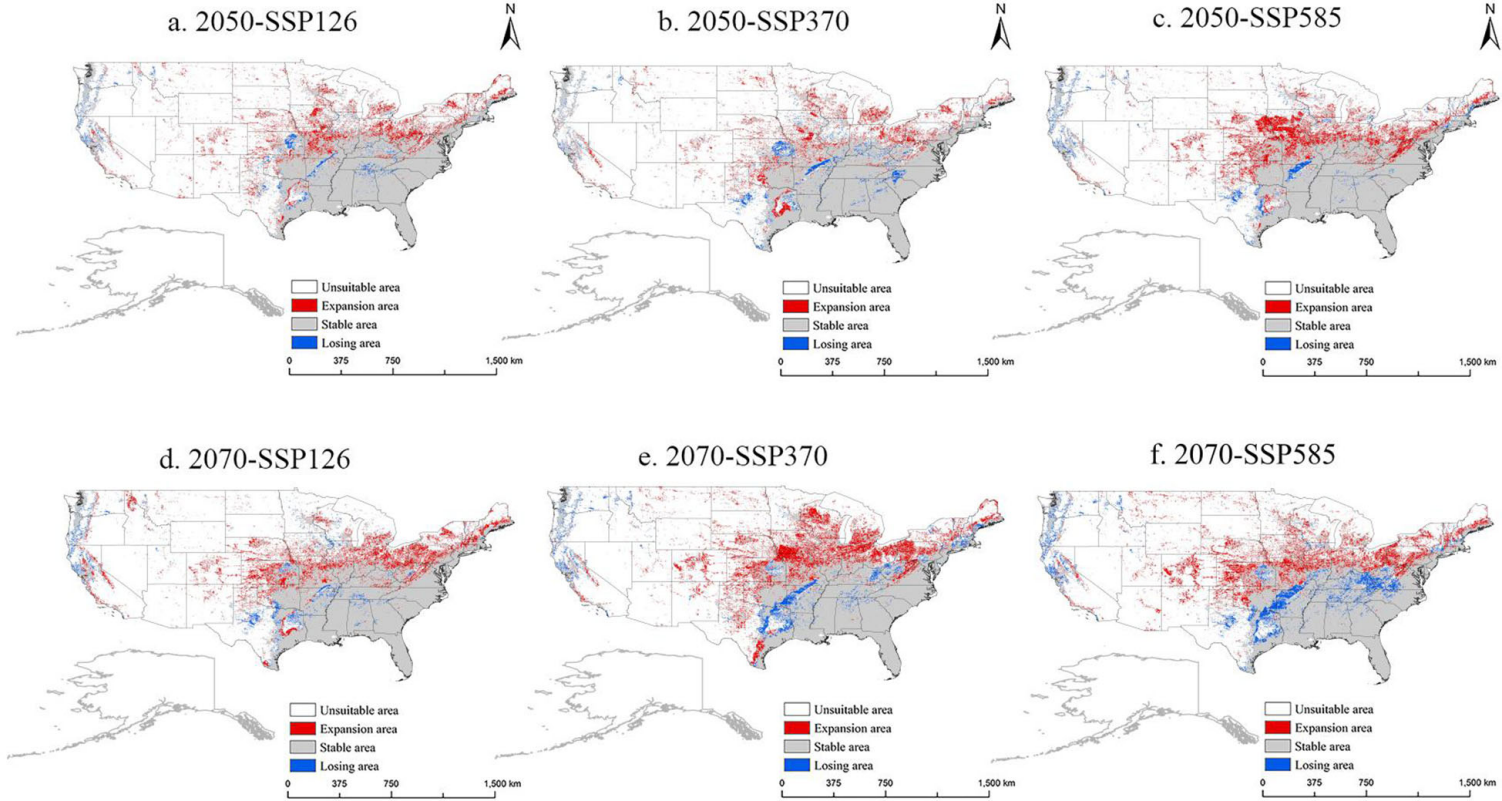
The potential distribution pattern of *M. grandiflora* in the USA under future climate scenarios (in the 2050s and 2070s). **(A)** 2050-SSP126, **(B)** 2050-SSP370, **(C)** 2050-SSP585, **(D)** 2070-SSP126, **(E)** 2070-SSP370, **(F)** 2070-SSP585. Note: White, red, gray, and blue represent non-suitable area, expansion area, stable area, and losing area, respectively.

For the USA, the future potential distribution trend of *M. grandiflora* is presented in **Figure 9**. With global climate change, *M. grandiflora* shows a migration toward northern regions, similar to the diffusion trend observed in China. In the 2050-SSP585 scenario, there is significant expansion into states such as Iowa, Kansas, Missouri, and Nebraska in the northwest. By the 2070s, the expansion is even greater, especially under the 2070-SSP370 scenario, with notable growth in western regions (Colorado, southern Nebraska, and Texas) and northern regions (Iowa, Wisconsin, southern Michigan, central Ohio, Pennsylvania, New York, Vermont, New Hampshire, and southern Maine). Areas of loss primarily appear along the southeastern and western coastlines. In 2050, the suitable area is projected to expand from USDA Cold Hardiness Zones 5b–8b. By contrast, in 2070, the expansion will primarily occur within USDA Cold Hardiness Zones 3a–11a, while the areas of loss will be concentrated in Zones 6b10a in the southeastern USA and Zones 8a–10b along the western coastlines.

## 4 Discussion

Since the twentieth century, the continuous accumulation of greenhouse gases has led to intensified global warming and rapid decline in global biodiversity (Omann et al., 2009; Raven and Wagner, 2021). Studies on the relationship between climate and plants has gradually become a hot topic in global change ecology. Understanding the changes in potential distribution and dynamic niche of plants under climate change helps to introduce plants reasonably and protect ecological resources (Piao et al., 2019). Our study mainly investigated the effects of five categories environmental variables on the distribution patterns of *M. grandiflora* and the dynamic changes of its ecological niche.

### 4.1 The creativity in the process of SDM construction

First, in order to fit the appropriate SDMs, Pearson correlation analysis and variance inflation factor (VIF) were used to reduce spatial autocorrelation among environmental variables (Keser et al., 2012). In addition, a buffer zone with a radius of 150 km was established based on GIS tools and ENM tools to prevent overfitting and model redundancy (Hengl et al., 2018), thus improving the accuracy of experiments in the specified study area. Subsequently, an ensemble model was constructed using qualified single models, greatly improving the accuracy of SDMs and better fitting the characteristics of the target species (Hao et al., 2019). Furthermore, to minimize errors and subjective outcomes, we utilized 5,000–10,000 points randomly generated by the Wallace package as the environmental variable background instead of the entire study area (Fuller et al., 2018).

### 4.2 The limitation of a single model and the necessity of a combination model

We used the Biomod2 package of SDM modeling *for M. grandiflora*, which ensemble multiple models to enhance prediction reliability and reduce individual model biases. This approach is crucial for adaptive management and conservation strategies, particularly under changing climatic conditions (Rodriguez et al., 2024; Wu H.-Y. et al., 2024). The models used in our study have demonstrated high reliability, with a higher AUC and TSS, indicating the robustness of the ensemble model.

### 4.3 Environmental explanations for the potential distribution of *M. grandiflora*

Through comparing the dynamic realized ecological niche of *M. grandiflora*, not only the predictive ability of the model for the distribution expansion of *M. grandiflora* is improved but also the influencing variables of its realized ecological niche were verified. Our study found that besides bioclimatic variables, temperature and precipitation were the key limiting variables for the dynamic ecological niche of *M. grandiflora*. *M. grandiflora* also grows in rich, loamy, and moist soils along streams and swamps in the Coastal Plain. It is also found in mesic upland areas where occurrences of fire are infrequent; therefore, other environmental variables such as solar radiation, water pH, soil texture, and NDVI should not be ignored. The results of the ensemble model (EM) stated that solar radiation, the human impact, precipitation, temperatures, potential evaporation volume, altitude, etc., played vital roles in the dispersal and spread of *M. grandiflora*. This is consistent with previous research results, indicating that *M. grandiflora* in the south grew in warm temperate to subtropical climates. The frost-free period is at least 210 days, exceeding 240 days in most distribution areas. The average temperature in January in coastal areas is 9°–12°C (49°F–54°F) in South Carolina and Georgia and 11°C–21°C (52°F–70°F) in Florida. The average temperature in July in coastal areas is 27°C (80°F). Annual precipitation ranges from 1,020 mm to 1,270 mm (40–50 in.) in the northeastern part of the distribution range and from 1,270 mm to 1,520 mm (50–60 in.) in other areas. A small area along the Gulf Coast receives annual precipitation of 1,520–2,030 mm (60–80 in.). The summer is typically the wettest and the fall is the driest in the Atlantic coastal plain region. Summer droughts are regular in the western part of the distribution range (Area, 2011; Rao and Davis, 1982).

### 4.4 Climatic niche overlap, equivalency, and similarity

The ecological niche defines the necessary environment conditions for the survival space of *M. grandiflora*. In addition to the main influencing variables such as temperature and precipitation, variables such as soil characteristics, solar radiation, and topographical variables also affected the ecological niche condition and expansion trends of *M. grandiflora*. Under the background of economic globalization, human activities are more frequent, and intentional or unintentional introduction and cultivation promote *M. grandiflora* to adapt to new climatic conditions. Although the equivalency test showed that niche morphology, size, and species distribution density of *M. grandiflora* in China and the USA were not equivalent, the similarity test and the higher ecological niche stability index proved that the growth climate conditions of *M. grandiflora* in China are generally similar to those in the United States (Mohamed et al., 2009).

### 4.5 Recommendations for cultivation and re-introduction of *M. grandiflora*

Our study found that both China and USA have more similar temperature changes and precipitation conditions. It is reasonable to infer that under future climate change, the potential distribution pattern changes in this species in its native USA, and its introduced country, China, will show similar expansion trends, including expansion to the northwest and northeast and contraction in the south. This suggests that *M. grandiflora* had much stronger cold and drought resistance (Clark et al., 1981; Delcourt and Delcourt, 1977). First, *M. grandiflora* exhibits a strong dependence on solar radiation. To mitigate excessive shading in densely vegetated areas, selective canopy thinning and pruning should be employed. These practices ensure that *M. grandiflora* receives adequate sunlight, promoting optimal photosynthesis and growth. Second, to safeguard the habitat of *M. grandiflora*, it is critical to restrict nearby infrastructure development, transportation expansion, and the use of chemical pollutants. These anthropogenic factors pose significant risks to the natural environment of *M. grandiflora*, and minimizing their impact is essential for the conservation of this species. Third, to establish a comprehensive hydrological monitoring system within the habitat of *M. grandiflora* is imperative for tracking precipitation patterns and soil moisture levels, where variations in rainfall may affect the water availability of *M. grandiflora*.

### 4.6 Strengths and limitations of the study

Despite the findings of our research on the ecological niche shift and potential distribution expansion trends of *M. grandiflora* and the high credibility of the model predictions, several uncertain variables also influenced the natural distribution pattern of this species. These variables included natural geographical barriers, adverse impacts of insects, species interactions, land use, model lags, and future climate normative policies. Further investigation is required to examine the pertinent principals.

## 5 Conclusions

In this paper, we aimed to clarify potential distribution changes in *M. grandiflora* between its native range (USA) and its introduced range (China) and the response of the ecological niche to environmental variables. Three ensemble models were adopted to predict the potential suitable habitat of *M. grandiflora* under three climate scenarios (SSP1-2.6, SSP3-7.0, and SSP5-8.5) in future periods (2050s and 2070s) and predict variation in its spatial pattern under climate change of the twenty-first century. Our research mainly found that (1) *M. grandiflora* exhibits similar distribution and dispersal trends in both its native USA and introduced China, with expansion towards the northeast and northwest under climate change, while shrinking in certain southern regions. (2) *M. grandiflora* is sensitive to environmental variables such as temperature, precipitation, water pH, and solar radiation, particularly favoring areas with lower sunshine in May and higher NDVI. (3) The high ecological niche unfilling index and similarity between the native and introduced regions suggest that *M. grandiflora* will likely continue expanding in areas with comparable climatic conditions. Overall, the higher ecological niche unfilling index and the similarity between the ecological niches of the native and introduced area indicated that *M. grandiflora* should continue to expand into introduced areas with similar climatic conditions with its native areas. The results of our study provide valuable insights into the potential distribution patterns and underlying factors driving the introduction of *M. grandiflora*. We propose that future research should incorporate more field data and experimental studies on the cold resistance of different *M. grandiflora* cultivars. This approach will help validate the model predictions and provide more detailed insights into the adaptability of *M. grandiflora* across various regions. In summary, the analysis of the environmental characteristics, ecological niche shift, and suitable areas of *M. grandiflor*a provides scientific references for plant protection, landscape use, and sustainable utilization, and serves as a valuable scientific reference for the prevention and management of biological introductions. Our method could also be applied to study other similar species of *Magnoliaceae*.

## Data Availability

The original contributions presented in the study are included in the article/**Supplementary Material**. Further inquiries can be directed to the corresponding author.
