# Supplementary material for: Analyzing the distribution patterns and dynamic niche of Magnolia grandiflora L. in the United States and China in response to climate change
Source: Front Plant Sci. 2024 Oct 22;15:1440610. doi: 10.3389/fpls.2024.1440610 (PMC11534871; doi:10.3389/fpls.2024.1440610)
Supplement: Supplementary file 2 [file Table2.docx]

**Supplementary Figures**


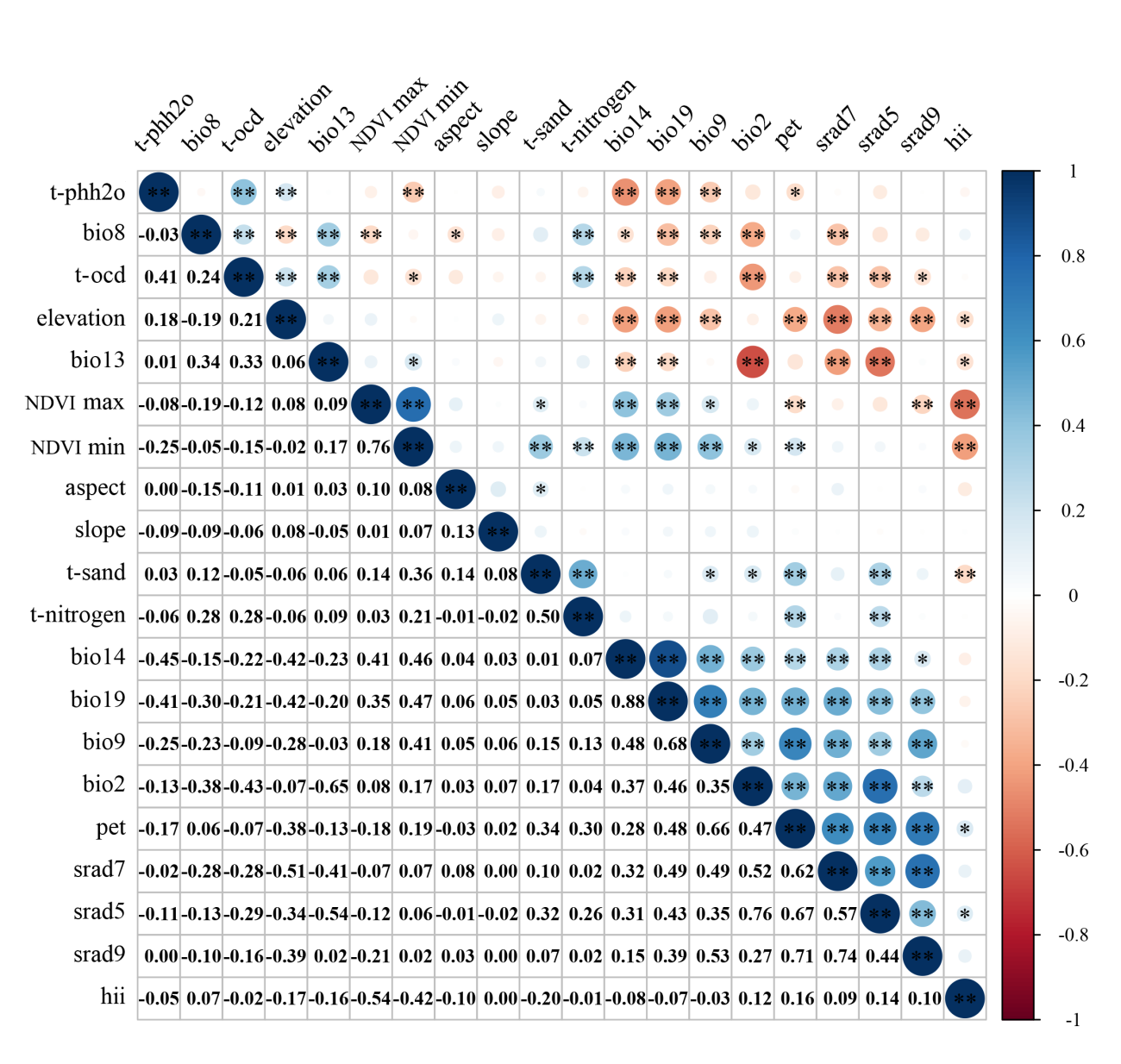


**Figure. S1** The hotplot of environment variables of pearson correlation analysis

Note: bio8 is Mean Temperature of Wettest Quarter; t-ocd is Organic carbon density; ele is elevation; bio13 is Precipitation of Wettest Month; NDVI-max is average maximum normalized difference vegetation index; min_clip is average minimum normalized difference vegetation index; bio14 is Precipitation of Driest Month; bio19 is Precipitation of Coldest Quarter; bio9 is Mean Temperature of Driest Quarter; bio2 is Mean Diurnal Range; pet is Potential Evapo-Transpiration; srad7 is Solar radiation in July; srad5 is Solar radiation in May; srad9 is Solar radiation in September; hii is Human Influence Index.
